# How leukocytes trigger opening and sealing of gaps in the endothelial barrier

**DOI:** 10.12688/f1000research.9185.1

**Published:** 2016-09-14

**Authors:** Debashree Goswami, Dietmar Vestweber

**Affiliations:** 1 Max Planck Institute for Molecular Biomedicine, Münster, Germany

**Keywords:** Leukocytes, endothelial barrier, transmigration, diapedesis

## Abstract

The entry of leukocytes into tissues requires well-coordinated interactions between the immune cells and endothelial cells which form the inner lining of blood vessels. The molecular basis for recognition, capture, and adhesion of leukocytes to the endothelial apical surface is well studied. This review will focus on recent advances in our understanding of events following the firm interaction of leukocytes with the inner surface of the blood vessel wall. We will discuss how leukocytes initiate the transmigration (diapedesis) process, trigger the opening of gaps in the endothelial barrier, and eventually move through this boundary.

## Introduction

Leukocytes circulating in the bloodstream represent a reservoir of immune cells that are passive as long as they are circulating. To bring them into a position where they can perform their immune functions, they need to first exit the vascular system. This requires sophisticated mechanisms that allow the leukocytes to recognize injured or infected tissue areas from within the vasculature. Recognition goes hand in hand with adhesion to the luminal surface of endothelial cells. These initial events are mediated by cytokine-induced endothelial adhesion molecules, such as the selectins, that mediate the capturing and rolling of leukocytes at the vessel wall. Selectins and chemokines presented on the endothelial cell surface trigger the activation of leukocyte integrins, which initiates leukocyte arrest and supports the crawling to appropriate exit sites. This well-studied multi-step process of leukocyte docking has been described in several excellent reviews ^1– 5^.

Here, we will focus on recent advances in our understanding of the subsequent steps of leukocyte extravasation, which are less well understood. For more extended discussions, the reader is also referred to some other recently published excellent reviews ^6– 9^. We will first give a short overview about endothelial adhesion receptors and signaling processes that support the diapedesis process. Considering this, we will then discuss what is currently known about the following questions: what determines the exit sites where the transmigration (diapedesis) process occurs and how do leukocytes recognize such sites? Which routes do leukocytes take to transmigrate through the endothelial barrier—the paracellular pathway through junctions or the transcellular route through the body of an endothelial cell—and what determines which route is taken? How are paracellular or transcellular gaps or pores through the endothelial barrier formed? How do leukocytes and endothelial cells maintain the barrier integrity and prevent plasma leakage during the diapedesis process?

## Endothelial membrane proteins that are involved in leukocyte diapedesis

**Figure 1.**
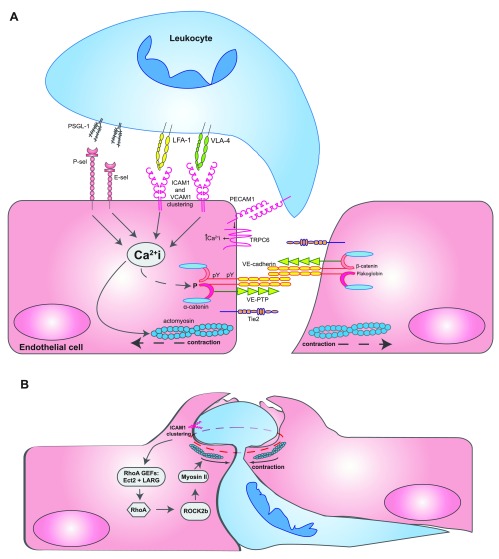
Opening and closing of endothelial junctions during diapedesis. ( **A**) Leukocytes interacting with several adhesion molecules on the endothelial cell surface trigger Ca ^2+^ signals inside endothelial cells, which are essential for leukocyte transmigration. It was reported that Ca ^2+^ signals triggered by the apical adhesion molecules were initiated by stores from the endoplasmic reticulum but that PECAM-1 Ca ^2+^ transients occurred rather local at transmigration sites through the TRPC6 channel. Ca ^2+^ signals trigger the activation of actomyosin-mediated pulling on endothelial junctions, influence the phosphorylation of components of the VE-cadherin-catenin complex, and trigger the recycling of the lateral border recycling compartment (LBRC) vesicle compartment. For a more detailed depiction of intracellular signaling steps, the reader is referred to recent reviews ^7– 9^. ( **B**) When leukocytes have already transmigrated more than halfway through the site of diapedesis, RhoA-mediated signaling triggered by the RHO guanine nucleotide exchange factors (GEFs) Ect2 and LARG stimulates ROCK2b, which activates actomyosin-based forces that support pore confinement, which leads to closure of the diapedesis pore ^87^. Abbreviations: ICAM1, intercellular adhesion molecule 1; LFA-1, lymphocyte function-associated antigen 1; PECAM-1, platelet and endothelial cell adhesion molecule 1; PSGL-1, P-selectin glycoprotein ligand-1; TRPC6, transient receptor potential canonical-6; VCAM1, vascular cell adhesion molecule 1; VE-cadherin, vascular endothelial cadherin; VE-PTP, vascular endothelial protein tyrosine phosphatase; VLA-4, very late antigen-4.

After capturing, rolling, and arrest, leukocytes crawl on the endothelial surface until they start to diapedese through the endothelial barrier. Arrest and crawling are mediated mainly by the β2-integrins LFA-1 (α _L_β _2_) and Mac-1 (α _M_β _2_) and the β1-integrin VLA-4 (α _4_β _1_) on leukocytes, of which the first two bind to endothelial intercellular adhesion molecule 1 (ICAM-1) and the latter binds to vascular cell adhesion protein 1 (VCAM-1). These endothelial adhesion molecules also act as signaling receptors and are instrumental for the initiation of signaling events, which affect the actomyosin cytoskeleton as well as adhesive structures at endothelial junctions, thereby facilitating and enabling the transmigration process ( Figure 1A). The concept that leukocytes trigger endothelial cells to support leukocyte transmigration goes back to studies by the Silverstein lab, which showed that neutrophil binding to endothelial cells triggered a Ca ^2+^ signal inside endothelial cells, which was required for transmigration but not for leukocyte adhesion ^10^. Later, this was linked to the phosphorylation of myosin light chain and the induction of isometric tension in endothelial cells ^11^. The relevance of Ca ^2+^ transients for the diapedesis process was confirmed in several reports ^12, 13^, although differences were found depending on which type of leukocytes were analyzed and whether endothelial cells were activated with cytokines ^14^. ICAM-1 was identified as a receptor that triggered lymphocyte-induced Ca ^2+^ transients in endothelial cells ^13^, and E- and P-selectin and VCAM-1 were also reported to have this capacity ^15^. More recently, it was found that the Ca ^2+^ channel TRPC6 is responsible in endothelial cells for Ca ^2+^ transients that are induced by leukocytes and are required for transmigration ( Figure 1A) ^16^. Another ion channel in endothelial cells, which is important for the recruitment of T cells into the brain, is the TWIK-related potassium channel-1 (TREK-1) ^17^.

Other signaling steps that are triggered by ICAM-1 and VCAM-1 are summarized in excellent reviews ^18, 19^. Activation of Rho family kinases such as RhoG is involved in the stable adhesion of leukocytes to the endothelium ^20^. In addition, the activation of RhoA is involved in ICAM-1-dependent rearrangements of the actomyosin system, leading to mechanotransduction in endothelial cells, which supports leukocyte transmigration ^21– 24^. Stimulation of the activation of Src family kinases leads to tyrosine phosphorylation of cortactin, which is relevant for the clustering of ICAM-1 ^25– 27^. Furthermore, clustering of ICAM-1 modulates tyrosine phosphorylation of vascular endothelial cadherin (VE-cadherin) and associated catenins, which is important for the opening of endothelial junctions ^28– 30^.

Several endothelial membrane proteins support the transmigration of leukocytes through the endothelial cell barrier. Platelet and endothelial cell adhesion molecule 1 (PECAM-1) was the first adhesion molecule that was shown to be involved in this process ^31^ and was followed by many others such as the junctional adhesion molecules JAM-A, -B, and -C ^32– 35^; endothelial cell-selective adhesion molecule (ESAM) ^36^; CD99 ^37, 38^; CD99L2 ^39– 41^; and the nectin-related poliovirus receptor (PVR) ^42^. All of these proteins are enriched at endothelial cell contacts. Although not much is known about how they facilitate the diapedesis process, some of them were shown to act in a sequential manner. PECAM-1 was found to act before CD99 ^37^, whereas PVR functions after PECAM-1 and before the CD99 step ^42^. ICAM-2 was reported to support one of the earliest steps in the diapedesis process followed by JAM-A and then PECAM-1 ^43^. Interference with some of these receptors, such as PECAM-1, CD99, and CD99L2, led to the accumulation of leukocytes between the endothelium and the basement membrane, suggesting that these proteins were also involved in mechanisms that enable leukocytes to overcome the basement membrane ^44, 45^.

Mechanistically, it was suggested that a multi-vesicular compartment inside endothelial cells, the lateral border recycling compartment (LBRC), would support the diapedesis process, possibly by serving as a membrane reservoir that helps accommodate the body of the transmigrating leukocyte at gaps between endothelial cells ^46^. PECAM-1 was suggested to trigger the mobilization of this compartment to the plasma membrane, whereas crosslinking of CD99 would trigger a second wave of vesicle traffic to cell contacts ^47^. The latter process was linked to the interaction of CD99 with ezrin, soluble adenylyl cyclase, and protein kinase A (PKA) ^47^. Engagement of PECAM-1 by leukocytes was reported to cluster the Ca ^2+^ channel TRPC6 at sites of diapedesis, and TRPC6 was found to act downstream of PECAM-1, affecting the recycling of LBRC vesicles ^16^. This report also suggested that Ca ^2+^ signals triggered by PECAM-1 via TRPC6 were rather local and different from global cellular Ca ^2+^ signals that are found after leukocyte docking to endothelial cells, which are mediated by Ca ^2+^ store release from the endoplasmic reticulum ( Figure 1A).

## What determines the site of leukocyte diapedesis?

Crawling on the luminal surface of postcapillary venules was reported to help leukocytes find appropriate exit sites ^48^. Several mechanisms were recently discussed which could determine such exit sites and allow leukocytes to identify them. Since ICAM-2 seems to act at the most apical position of all diapedesis-supporting cell surface receptors ^43^, it might be a candidate for a receptor that could help leukocytes to identify endothelial cell contacts. ICAM-2 is diffusely expressed over the whole apical surface of endothelial cells in postcapillary venules but is enriched at endothelial cell contacts ^49^. Apical ICAM-2 supports as a ligand for Mac-1 neutrophil crawling and interfering with its function altered the stop-and-go intervals of leukocyte crawling ^49^. It is an attractive speculation that the increased expression levels of ICAM-2 at cell contacts might influence leukocytes in their decision to stop and start diapedesing.

Recently, platelets were described as pathfinders for leukocyte extravasation. It was shown that platelets adhered under inflammatory conditions at endothelial junctions in the smallest venular microvessels and captured neutrophils via CD40-CD40L/CD154-dependent interactions ^50^. Intravascularly adherent platelets and neutrophils, in turn, recruited inflammatory monocytes to these sites of extravasation. These interactions required the interaction of P-selectin with leukocyte PSGL-1, which contributed to the activation of leukocyte integrins. Blockade of these multi-cellular interactions reduced leukocyte extravasation. These findings provide mechanistic understanding for the previously well-documented important contribution of platelets to leukocyte extravasation ^51– 54^.

Another aspect that may determine a transmigration site is the stiffness of endothelial cells. It was found that endothelial stiffness supported the spreading and transmigration of neutrophils. A gradient of increasing stiffness (measured by atomic force microscopy) from the center to the periphery of endothelial cells drove crawling neutrophils toward cell junctions, promoting transmigration through a paracellular route ^55^. In contrast, another study showed that lymphocytes transmigrated preferentially through local sites of reduced endothelial cell stiffness, which were characterized by low levels of F-actin and were also found at sites of transcellular migration ^56^. Interestingly, long time exposure of endothelial cells to flow stabilizes junctions and this increased the fraction of lymphocytes that transmigrated through a transcellular route ^56^.

## Which routes do leukocytes take through the endothelial barrier?

Leukocytes transmigrate through a paracellular route, which requires the opening of endothelial junctions, and through a transcellular route, which does not require junction opening but is often close to junctions. Both processes have been well documented *in vitro* and *in vivo*. Direct analysis by intravital three-dimensional video microscopy showed that 90% of extravasating neutrophils use the paracellular route and that only 10% use the transcellular route, and this was found under different inflammatory stimuli ^35^. In line with this, stabilizing endothelial junctions in genetically modified mice by replacing endogenous VE-cadherin with a VE-cadherin-α-catenin fusion construct strongly inhibits neutrophil extravasation in lung and cremaster and lymphocyte recruitment into inflamed skin by 70 to 80% ^57^. *In vitro*, more than 90% of neutrophils, monocytes, and lymphocytes transmigrate through human umbilical vein endothelial cells (HUVECs) via the paracellular route ^58^. The low efficiency of the transcellular route is enhanced up to 30% for leukocyte transmigration through cultured microvascular endothelial cells ^59^. In addition, higher expression levels of ICAM-1 on endothelial cells correlated with a higher percentage of transcellular events ^60, 61^. The latter study also showed that higher ICAM-1 expression levels correlated with reduced crawling distances of cells under flow. Interestingly, this study also reported that the few lymphocytes that still transmigrated through endothelial cells lacking ICAM-1 and ICAM-2 were unable to crawl prior to diapedesis and used exclusively the transcellular route ^61^. Thus, it is tempting to speculate that leukocytes, which are hindered to reach their optimal site of exit at junctions or which reside longer than normal at a site on the apical endothelial surface, may tend to use a transcellular route. In agreement with this concept, interfering with the integrin Mac-1 on neutrophils, which inhibited crawling, enhanced the percentage of transcellular migration *in vivo* ^48^. Likewise, T cells deficient for the Rac1 guanine nucleotide exchange factor (GEF) Tiam1 showed reduced crawling and used the transcellular diapedesis route with enhanced efficiency ^62^.

## How are gaps in the endothelial barrier opened during leukocyte diapedesis?

Paracellular diapedesis of leukocytes requires the opening of endothelial junctions. VE-cadherin is an important player in this process since antibodies against VE-cadherin can enhance leukocyte extravasation *in vivo* ^63^ and enhancing the adhesive activity of VE-cadherin by replacing it with a VE-cadherin-α-catenin fusion protein in knock-in mice strongly inhibits leukocyte extravasation in several tissues ^57^. *In vitro* studies revealed that leukocyte-triggered clustering of ICAM-1 modulated tyrosine phosphorylation of VE-cadherin, which was linked to the opening of junctions and transmigration efficiency ^28, 29^. The transmigration of B16 melanoma cells through cultured endothelial cells was reported to require endothelial FAK activity, which triggered the phosphorylation of Y658 of VE-cadherin ^64^. *In vivo* studies with mice carrying tyrosine/phenylalanine (Y/F) point mutations in Y731 or Y685 of VE-cadherin revealed that leukocyte-induced dephosphorylation of Y731 was required for neutrophil extravasation *in vivo* ^30^ ( Figure 1A). This dephosphorylation was dependent on the phosphatase SHP-2, which led to enhanced endocytosis of VE-cadherin. Interestingly, stimulation of vascular permeability by inflammatory mediators did not require Y731 of VE-cadherin but was dependent on the upregulation of pY685 ^30^. This revealed that opening of endothelial junctions *in vivo* requires the modulation of VE-cadherin tyrosine phosphorylation; however, different tyrosines are addressed in the context of vascular permeability induction and leukocyte extravasation. It is attractive to speculate that the dephosphorylation of Y731 on VE-cadherin initiates a more robust opening of junctions, which allows the passage of transmigrating leukocytes, whereas the induction of Y685 phosphorylation leads to a more subtle destabilization of endothelial contacts, which is sufficient for plasma leaks.

An important regulator of endothelial junction integrity is the endothelial-specific receptor-type tyrosine phosphatase called vascular endothelial protein tyrosine phosphatase (VE-PTP), which associates with VE-cadherin and supports its adhesive activity ^65^, in part by inhibiting tyrosine phosphorylation of plakoglobin ^66^. Docking of leukocytes to endothelial cells as well as stimulation of endothelial cells with vascular endothelial growth factor (VEGF) or histamine triggers rapid dissociation of VE-PTP from VE-cadherin ^66^. Each of these different stimuli triggers the same signaling cascade that leads to VE-PTP/VE-cadherin dissociation ^67^. It could be demonstrated that this dissociation is necessary for the induction of vascular permeability and for inflammation-induced neutrophil extravasation *in vivo* ^68^. Interestingly, VE-PTP is able to dephosphorylate Y685 but not Y731 of VE-cadherin ^30^. Thus, it is likely that VE-PTP affects the regulation of vascular permeability via Y685 of VE-cadherin but that the substrate relevant for the role of VE-PTP in leukocyte diapedesis is probably plakoglobin ^30, 66^. A role of VE-PTP for the control of endothelial cell integrity *in vivo* was also shown in a recent study that reported the induction of VE-PTP by hypoxia via HIF2α ^69^. Besides VE-cadherin, VE-PTP also associates with the tyrosine kinase receptor Tie-2 ^70^, which is important for vascular remodeling in embryonic development ^71^ ( Figure 1A). Interfering with VE-PTP in various ways leads to the stabilization of endothelial junctions ^72^. This effect is mediated by Tie-2. It also attenuates neutrophil recruitment into inflamed tissue by blocking actomyosin pulling forces on endothelial junctions ^73^.

In addition to counteracting the function of VE-cadherin, leukocytes also need to trigger mechanisms that actively open gaps for extravasation. As mentioned above, clustering of ICAM-1 triggers endothelial Ca ^2+^ signals and phosphorylation of myosin light chain kinase (MLCK) ^11^, which has been implicated in the modulation of endothelial junctions ^74^. Inhibition of MLCK prevents neutrophil diapedesis ^75^, and also Rho kinase was shown to be involved ^76^. In agreement with this, the endothelial microfilament system is required for the transmigration of monocytes ^77^. These reports are in line with our previous findings that showed that stimulation of the endothelial tyrosine kinase receptor Tie-2 can inhibit neutrophil recruitment to endotoxin-stimulated mouse lungs ^73^. These effects were even observed in mice conditionally gene-inactivated for VE-cadherin. Since Tie-2 activation was found to reduce radial stress fiber formation and blocked MLC phosphorylation by a Rap-1- and Rac-1-dependent mechanism, these results suggest that leukocyte extravasation opens endothelial junctions by a two-step mechanism: downregulation of VE-cadherin function and active, actomyosin-mediated pulling on endothelial cell contacts ^73^.

For the transcellular migration pathway, it is less clear how the required transcellular gaps are formed. It was shown that membrane trafficking-related proteins such as vasodilator-stimulated phosphoprotein (VASP) and caveolin are involved in the formation of the transcellular pathway ^78^. Furthermore, it was suggested that regulated membrane fusion events requiring NSF (N-ethylmaleimide sensitive factor) and SNARE (soluble NSF attachment protein receptor) complex proteins would be required in endothelial cells for transcellular leukocyte diapedesis ^59^. Endothelial cells are often rather flat in large areas of their bodies, with a thickness of no more than 1 μm at their edges. It may be possible that at certain sites the apical and the basal membranes fuse directly. Alternatively, intracellular vesiculo-vacuolar organelles inside endothelial cells might fuse with each other and with the apical and basal membranes to form a short “channel” that could accommodate transmigrating leukocytes on a transcellular route ^79^. It is interesting that several of the adhesion receptors that are involved in the paracellular transmigration of leukocytes, such as PECAM-1, CD99, and JAM-A, were also found encircling transcellularly migrating leukocytes ^59, 80^. Antibodies against these antigens also interfered with the transcellular transmigration, although the role of these antigens in transcellular migration is unknown at present.

## How are leaks prevented when leukocytes exit circulation?

Two hallmarks of inflammation are leukocyte extravasation and plasma leaks. Since they are often coinciding, it was debated for a long time whether leukocyte diapedesis would be the cause for the increase in vascular permeability. Arguments against this are based on cases where both events were documented at different sites in the vascular bed of inflamed tissues ^81– 84^ and at different times during the inflammatory process ^85, 86^. Thus, mechanisms must be in place to ensure a tight endothelial barrier, although leukocytes open endothelial junctions and transmigrate through them. Recently, it was reported that such a mechanism is based on ICAM-1-triggered activation of RhoA, mediated by the RHO GEFs Ect2 and LARG ( Figure 1B). This stimulates the activation of the kinase ROCK2b, which in turn activates acto-myosin-based endothelial pore confinement ^87^.

Interestingly, this study showed that interfering with endothelial RhoA *in vitro* and *in vivo* caused neutrophil-induced vascular leaks but did not inhibit the transmigration of neutrophils and this is in agreement with other *in vitro* studies ^58^. Thus, RhoA activity in endothelial cells is redundant for leukocyte transmigration, which is in contrast to other reports discussed above, which suggests RhoA triggered actomyosin pulling on endothelial junctions as a facilitator of the diapedesis process ^21, 76^. Although this is clearly a novel view of the role of RhoA in the diapedesis process, it does not argue against the concept of radial actomyosin stress fibers as being responsible for opening junctions by exerting pulling forces. It is possible that other GTPases are responsible for the activation of non-muscle myosin II. Furthermore, calcium/calmodulin is able to activate MLCK; thus, leukocyte-induced activation of non-muscle myosin II would not necessarily require RhoA.

A more dramatic type of leak formation that is linked to the extravasation of neutrophils is visible only under thrombocytopenic conditions (that is, when platelets are depleted). It was shown that, under such conditions, local bleedings (petechiae) occur at sites of local inflammation ^88^. Recently, it was shown that it is the diapedesis of neutrophils which triggers the exit of erythrocytes under conditions of thrombocytopenia ^54^. This implies that platelets prevent the exit of erythrocytes at sites of neutrophil extravasation. Mechanistically, it was shown that platelets require the ITAM receptors CLEC2 and GPVI ^89^ for this protective effect against erythrocyte leaks and it was suggested that single platelets seal neutrophil-induced vascular breaches ^90^.

## Outlook/future directions

Recent years have provided the first mechanistic insights into the process of leukocyte diapedesis through the endothelial barrier. Although several cell surface adhesion receptors have been identified at endothelial cell contacts that are involved in the transmigration process (and not in leukocyte capturing and docking), the first signaling mechanisms triggered by PECAM-1 and by CD99 were identified only recently. It will be a major goal in the future to elucidate the potential roles of the various receptors for the following functions: signaling to leukocytes to convey the information that an exit site has been reached; opening and possibly sealing of gaps in the endothelial barrier; participation in leukocyte migration; and facilitating mechanisms that enable leukocytes to overcome the basement membrane. The last step of migration through the basement membrane is still enigmatic ^91– 93^. It is an interesting concept that the composition of the basement membrane and low expression regions of certain components of the basement membrane represent preferred sites of transmigration ^94, 95^, which may even have an impact on the ability of associated endothelial cells to serve as preferred entry sites. Likewise, pericytes could have guiding effects in this respect ^96^. Finally, it was recently discovered that the transmigration process is not always unidirectional and that under certain conditions neutrophils can also revert the direction and move back into the circulation ^34, 97, 98^. It will be interesting to elucidate the physiological and pathophysiological relevance of this process.

## References

[1] Butcher EC: Leukocyte-endothelial cell recognition: three (or more) steps to specificity and diversity. *Cell.* 1991;67(6):1033–6. 10.1016/0092-8674(91)90279-8 1760836

[2] Springer TA: Traffic signals on endothelium for lymphocyte recirculation and leukocyte emigration. *Annu Rev Physiol.* 1995;57:827–72. 10.1146/annurev.ph.57.030195.004143 7778885

[3] Ley K Laudanna C Cybulsky MI: Getting to the site of inflammation: the leukocyte adhesion cascade updated. *Nat Rev Immunol.* 2007;7(9):678–89. 10.1038/nri2156 17717539

[4] Alon R Feigelson SW: Chemokine-triggered leukocyte arrest: force-regulated bi-directional integrin activation in quantal adhesive contacts. *Curr Opin Cell Biol.* 2012;24(5):670–6. 10.1016/j.ceb.2012.06.001 22770729

[5] McEver RP: Selectins: initiators of leucocyte adhesion and signalling at the vascular wall. *Cardiovasc Res.* 2015;107(3):331–9. 10.1093/cvr/cvv154 25994174 PMC4592324

[6] Hordijk PL: Recent insights into endothelial control of leukocyte extravasation. *Cell Mol Life Sci.* 2016;73(8):1591–608. 10.1007/s00018-016-2136-y 26794844 PMC11108429

[7] Vestweber D: How leukocytes cross the vascular endothelium. *Nat Rev Immunol.* 2015;15(11):692–704. 10.1038/nri3908 26471775

[8] Muller WA: The regulation of transendothelial migration: new knowledge and new questions. *Cardiovasc Res.* 2015;107(3):310–20. 10.1093/cvr/cvv145 25987544 PMC4592322

[9] Nourshargh S Alon R: Leukocyte migration into inflamed tissues. *Immunity.* 2014;41(5):694–707. 10.1016/j.immuni.2014.10.008 25517612

[10] Huang AJ Manning JE Bandak TM: Endothelial cell cytosolic free calcium regulates neutrophil migration across monolayers of endothelial cells. *J Cell Biol.* 1993;120(6):1371–80. 10.1083/jcb.120.6.1371 8449983 PMC2119745

[11] Hixenbaugh EA Goeckeler ZM Papaiya NN: Stimulated neutrophils induce myosin light chain phosphorylation and isometric tension in endothelial cells. *Am J Physiol.* 1997;273(2 Pt 2):H981–8. 9277518 10.1152/ajpheart.1997.273.2.H981

[12] Su WH Chen HI Huang JP: Endothelial [Ca ^2+^] _i_ signaling during transmigration of polymorphonuclear leukocytes. *Blood.* 2000;96(12):3816–22. 11090065

[13] Etienne-Manneville S Manneville JB Adamson P: ICAM-1-coupled cytoskeletal rearrangements and transendothelial lymphocyte migration involve intracellular calcium signaling in brain endothelial cell lines. *J Immunol.* 2000;165(6):3375–83. 10.4049/jimmunol.165.6.3375 10975856

[14] Kielbassa-Schnepp K Strey A Janning A: Endothelial intracellular Ca ^2+^ release following monocyte adhesion is required for the transendothelial migration of monocytes. *Cell Calcium.* 2001;30(1):29–40. 10.1054/ceca.2001.0210 11396985

[15] Lorenzon P Vecile E Nardon E: Endothelial cell E- and P-selectin and vascular cell adhesion molecule-1 function as signaling receptors. *J Cell Biol.* 1998;142(5):1381–91. 10.1083/jcb.142.5.1381 9732297 PMC2149355

[16] Weber EW Han F Tauseef M: TRPC6 is the endothelial calcium channel that regulates leukocyte transendothelial migration during the inflammatory response. *J Exp Med.* 2015;212(11):1883–99. 10.1084/jem.20150353 26392222 PMC4612081

[17] Bittner S Ruck T Schuhmann MK: Endothelial TWIK-related potassium channel-1 (TREK1) regulates immune-cell trafficking into the CNS. *Nat Med.* 2013;19(9):1161–5. 10.1038/nm.3303 23933981

[18] Fernandez-Borja M van Buul JD Hordijk PL: The regulation of leucocyte transendothelial migration by endothelial signalling events. *Cardiovasc Res.* 2010;86(2):202–10. 10.1093/cvr/cvq003 20068003

[19] van Buul JD Kanters E Hordijk PL: Endothelial signaling by Ig-like cell adhesion molecules. *Arterioscler Thromb Vasc Biol.* 2007;27(9):1870–6. 10.1161/ATVBAHA.107.145821 17585068

[20] van Buul JD Allingham MJ Samson T: RhoG regulates endothelial apical cup assembly downstream from ICAM1 engagement and is involved in leukocyte trans-endothelial migration. *J Cell Biol.* 2007;178(7):1279–93. 10.1083/jcb.200612053 17875742 PMC2064659

[21] Etienne S Adamson P Greenwood J: ICAM-1 signaling pathways associated with Rho activation in microvascular brain endothelial cells. *J Immunol.* 1998;161(10):5755–61. 9820557

[22] Millán J Ridley AJ: Rho GTPases and leucocyte-induced endothelial remodelling. *Biochem J.* 2005;385(Pt 2):329–37. 10.1042/BJ20041584 15496138 PMC1134702

[23] Adamson P Etienne S Couraud PO: Lymphocyte migration through brain endothelial cell monolayers involves signaling through endothelial ICAM-1 via a rho-dependent pathway. *J Immunol.* 1999;162(5):2964–73. 10072547

[24] Lessey-Morillon EC Osborne LD Monaghan-Benson E: The RhoA guanine nucleotide exchange factor, LARG, mediates ICAM-1-dependent mechanotransduction in endothelial cells to stimulate transendothelial migration. *J Immunol.* 2014;192(7):3390–8. 10.4049/jimmunol.1302525 24585879 PMC3991232

[25] Durieu-Trautmann O Chaverot N Cazaubon S: Intercellular adhesion molecule 1 activation induces tyrosine phosphorylation of the cytoskeleton-associated protein cortactin in brain microvessel endothelial cells. *J Biol Chem.* 1994;269(17):12536–40. 7909803

[26] Yang L Kowalski JR Yacono P: Endothelial cell cortactin coordinates intercellular adhesion molecule-1 clustering and actin cytoskeleton remodeling during polymorphonuclear leukocyte adhesion and transmigration. *J Immunol.* 2006;177(9):6440–9. 10.4049/jimmunol.177.9.6440 17056576

[27] Schnoor M Lai FP Zarbock A: Cortactin deficiency is associated with reduced neutrophil recruitment but increased vascular permeability *in vivo*. *J Exp Med.* 2011;208(8):1721–35. 10.1084/jem.20101920 21788407 PMC3149227

[28] Allingham MJ van Buul JD Burridge K: ICAM-1-mediated, Src- and Pyk2-dependent vascular endothelial cadherin tyrosine phosphorylation is required for leukocyte transendothelial migration. *J Immunol.* 2007;179(6):4053–64. 10.4049/jimmunol.179.6.4053 17785844

[29] Turowski P Martinelli R Crawford R: Phosphorylation of vascular endothelial cadherin controls lymphocyte emigration. *J Cell Sci.* 2008;121(Pt 1):29–37. 10.1242/jcs.022681 18096689 PMC3810954

[30] Wessel F Winderlich M Holm M: Leukocyte extravasation and vascular permeability are each controlled *in vivo* by different tyrosine residues of VE-cadherin. *Nat Immunol.* 2014;15(3):223–30. 10.1038/ni.2824 24487320

[31] Muller WA Weigl SA Deng X: PECAM-1 is required for transendothelial migration of leukocytes. *J Exp Med.* 1993;178(2):449–60. 10.1084/jem.178.2.449 8340753 PMC2191108

[32] Martìn-Padura I Lostaglio S Schneemann M: Junctional adhesion molecule, a novel member of the immunoglobulin superfamily that distributes at intercellular junctions and modulates monocyte transmigration. *J Cell Biol.* 1998;142(1):117–27. 10.1083/jcb.142.1.117 9660867 PMC2133024

[33] Nourshargh S Krombach F Dejana E: The role of JAM-A and PECAM-1 in modulating leukocyte infiltration in inflamed and ischemic tissues. *J Leukoc Biol.* 2006;80(4):714–8. 10.1189/jlb.1105645 16857733

[34] Bradfield PF Scheiermann C Nourshargh S: JAM-C regulates unidirectional monocyte transendothelial migration in inflammation. *Blood.* 2007;110(7):2545–55. 10.1182/blood-2007-03-078733 17625065 PMC1988941

[35] Woodfin A Voisin MB Beyrau M: The junctional adhesion molecule JAM-C regulates polarized transendothelial migration of neutrophils *in vivo*. *Nat Immunol.* 2011;12(8):761–9. 10.1038/ni.2062 21706006 PMC3145149

[36] Wegmann F Petri B Khandoga AG: ESAM supports neutrophil extravasation, activation of Rho, and VEGF-induced vascular permeability. *J Exp Med.* 2006;203(7):1671–7. 10.1084/jem.20060565 16818677 PMC2118342

[37] Schenkel AR Mamdouh Z Chen X: CD99 plays a major role in the migration of monocytes through endothelial junctions. *Nat Immunol.* 2002;3(2):143–50. 10.1038/ni749 11812991

[38] Bixel G Kloep S Butz S: Mouse CD99 participates in T-cell recruitment into inflamed skin. *Blood.* 2004;104(10):3205–13. 10.1182/blood-2004-03-1184 15280198

[39] Bixel MG Petri B Khandoga AG: A CD99-related antigen on endothelial cells mediates neutrophil but not lymphocyte extravasation *in vivo*. *Blood.* 2007;109(12):5327–36. 10.1182/blood-2006-08-043109 17344467

[40] Schenkel AR Dufour EM Chew TW: The murine CD99-related molecule CD99-like 2 (CD99L2) is an adhesion molecule involved in the inflammatory response. *Cell Commun Adhes.* 2007;14(5):227–37. 10.1080/15419060701755966 18163232

[41] Seelige R Natsch C März S: Cutting edge: Endothelial-specific gene ablation of CD99L2 impairs leukocyte extravasation *in vivo*. *J Immunol.* 2013;190(3):892–6. 10.4049/jimmunol.1202721 23293350

[42] Reymond N Imbert A Devilard E: DNAM-1 and PVR regulate monocyte migration through endothelial junctions. *J Exp Med.* 2004;199(10):1331–41. 10.1084/jem.20032206 15136589 PMC2211807

[43] Woodfin A Voisin MB Imhof BA: Endothelial cell activation leads to neutrophil transmigration as supported by the sequential roles of ICAM-2, JAM-A, and PECAM-1. *Blood.* 2009;113(24):6246–57. 10.1182/blood-2008-11-188375 19211506 PMC2699241

[44] Woodfin A Reichel CA Khandoga A: JAM-A mediates neutrophil transmigration in a stimulus-specific manner *in vivo*: evidence for sequential roles for JAM-A and PECAM-1 in neutrophil transmigration. *Blood.* 2007;110(6):1848–56. 10.1182/blood-2006-09-047431 17505016

[45] Bixel MG Li H Petri B: CD99 and CD99L2 act at the same site as, but independently of, PECAM-1 during leukocyte diapedesis. *Blood.* 2010;116(7):1172–84. 10.1182/blood-2009-12-256388 20479283

[46] Mamdouh Z Chen X Pierini LM: Targeted recycling of PECAM from endothelial surface-connected compartments during diapedesis. *Nature.* 2003;421(6924):748–53. 10.1038/nature01300 12610627

[47] Watson RL Buck J Levin LR: Endothelial CD99 signals through soluble adenylyl cyclase and PKA to regulate leukocyte transendothelial migration. *J Exp Med.* 2015;212(7):1021–41. 10.1084/jem.20150354 26101266 PMC4493416

[48] Phillipson M Heit B Colarusso P: Intraluminal crawling of neutrophils to emigration sites: a molecularly distinct process from adhesion in the recruitment cascade. *J Exp Med.* 2006;203(12):2569–75. 10.1084/jem.20060925 17116736 PMC2118150

[49] Halai K Whiteford J Ma B: ICAM-2 facilitates luminal interactions between neutrophils and endothelial cells *in vivo*. *J Cell Sci.* 2014;127(Pt 3):620–9. 10.1242/jcs.137463 24317296 PMC4007766

[50] Zuchtriegel G Uhl B Puhr-Westerheide D: Platelets Guide Leukocytes to Their Sites of Extravasation. *PLoS Biol.* 2016;14(5):e1002459. 10.1371/journal.pbio.1002459 27152726 PMC4859536

[51] Sreeramkumar V Adrover JM Ballesteros I: Neutrophils scan for activated platelets to initiate inflammation. *Science.* 2014;346(6214):1234–8. 10.1126/science.1256478 25477463 PMC4280847

[52] Rossaint J Spelten O Kässens N: Acute loss of renal function attenuates slow leukocyte rolling and transmigration by interfering with intracellular signaling. *Kidney Int.* 2011;80(5):493–503. 10.1038/ki.2011.125 21562471 PMC3156340

[53] Petri B Broermann A Li H: von Willebrand factor promotes leukocyte extravasation. *Blood.* 2010;116(22):4712–9. 10.1182/blood-2010-03-276311 20716766

[54] Hillgruber C Pöppelmann B Weishaupt C: Blocking neutrophil diapedesis prevents hemorrhage during thrombocytopenia. *J Exp Med.* 2015;212(8):1255–66. 10.1084/jem.20142076 26169941 PMC4516803

[55] Schaefer A Hordijk PL: Cell-stiffness-induced mechanosignaling - a key driver of leukocyte transendothelial migration. *J Cell Sci.* 2015;128(13):2221–30. 10.1242/jcs.163055 26092932

[56] Martinelli R Zeiger AS Whitfield M: Probing the biomechanical contribution of the endothelium to lymphocyte migration: diapedesis by the path of least resistance. *J Cell Sci.* 2014;127(Pt 17):3720–34. 10.1242/jcs.148619 25002404 PMC4150060

[57] Schulte D Küppers V Dartsch N: Stabilizing the VE-cadherin-catenin complex blocks leukocyte extravasation and vascular permeability. *EMBO J.* 2011;30(20):4157–70. 10.1038/emboj.2011.304 21857650 PMC3199392

[58] Carman CV Springer TA: A transmigratory cup in leukocyte diapedesis both through individual vascular endothelial cells and between them. *J Cell Biol.* 2004;167(2):377–88. 10.1083/jcb.200404129 15504916 PMC2172560

[59] Carman CV Sage PT Sciuto TE: Transcellular diapedesis is initiated by invasive podosomes. *Immunity.* 2007;26(6):784–97. 10.1016/j.immuni.2007.04.015 17570692 PMC2094044

[60] Yang L Froio RM Sciuto TE: ICAM-1 regulates neutrophil adhesion and transcellular migration of TNF-alpha-activated vascular endothelium under flow. *Blood.* 2005;106(2):584–92. 10.1182/blood-2004-12-4942 15811956 PMC1635241

[61] Abadier M Haghayegh Jahromi N Cardoso Alves L: Cell surface levels of endothelial ICAM-1 influence the transcellular or paracellular T-cell diapedesis across the blood-brain barrier. *Eur J Immunol.* 2015;45(4):1043–58. 10.1002/eji.201445125 25545837

[62] Gérard A van der Kammen RA Janssen H: The Rac activator Tiam1 controls efficient T-cell trafficking and route of transendothelial migration. *Blood.* 2009;113(24):6138–47. 10.1182/blood-2008-07-167668 19139083

[63] Gotsch U Borges E Bosse R: VE-cadherin antibody accelerates neutrophil recruitment *in vivo*. *J Cell Sci.* 1997;110(Pt 5):583–8. 9092940 10.1242/jcs.110.5.583

[64] Jean C Chen XL Nam JO: Inhibition of endothelial FAK activity prevents tumor metastasis by enhancing barrier function. *J Cell Biol.* 2014;204(2):247–63. 10.1083/jcb.201307067 24446483 PMC3897185

[65] Nawroth R Poell G Ranft A: VE-PTP and VE-cadherin ectodomains interact to facilitate regulation of phosphorylation and cell contacts. *EMBO J.* 2002;21(18):4885–95. 10.1093/emboj/cdf497 12234928 PMC126293

[66] Nottebaum AF Cagna G Winderlich M: VE-PTP maintains the endothelial barrier via plakoglobin and becomes dissociated from VE-cadherin by leukocytes and by VEGF. *J Exp Med.* 2008;205(12):2929–45. 10.1084/jem.20080406 19015309 PMC2585844

[67] Vockel M Vestweber D: How T cells trigger the dissociation of the endothelial receptor phosphatase VE-PTP from VE-cadherin. *Blood.* 2013;122(14):2512–22. 10.1182/blood-2013-04-499228 23908467

[68] Broermann A Winderlich M Block H: Dissociation of VE-PTP from VE-cadherin is required for leukocyte extravasation and for VEGF-induced vascular permeability *in vivo*. *J Exp Med.* 2011;208(12):2393–401. 10.1084/jem.20110525 22025303 PMC3256962

[69] Gong H Rehman J Tang H: HIF2α signaling inhibits adherens junctional disruption in acute lung injury. *J Clin Invest.* 2015;125(2):652–64. 10.1172/JCI77701 25574837 PMC4319409

[70] Fachinger G Deutsch U Risau W: Functional interaction of vascular endothelial-protein-tyrosine phosphatase with the angiopoietin receptor Tie-2. *Oncogene.* 1999;18(43):5948–53. 10.1038/sj.onc.1202992 10557082

[71] Winderlich M Keller L Cagna G: VE-PTP controls blood vessel development by balancing Tie-2 activity. *J Cell Biol.* 2009;185(4):657–71. 10.1083/jcb.200811159 19451274 PMC2711575

[72] Shen J Frye M Lee BL: Targeting VE-PTP activates TIE2 and stabilizes the ocular vasculature. *J Clin Invest.* 2014;124(10):4564–76. 10.1172/JCI74527 25180601 PMC4191011

[73] Frye M Dierkes M Küppers V: Interfering with VE-PTP stabilizes endothelial junctions *in vivo* via Tie-2 in the absence of VE-cadherin. *J Exp Med.* 2015;212(13):2267–87. 10.1084/jem.20150718 26642851 PMC4689167

[74] Garcia JG Verin AD Herenyiova M: Adherent neutrophils activate endothelial myosin light chain kinase: role in transendothelial migration. *J Appl Physiol (1985).* 1998;84(5):1817–21. 9572834 10.1152/jappl.1998.84.5.1817

[75] Saito H Minamiya Y Kitamura M: Endothelial myosin light chain kinase regulates neutrophil migration across human umbilical vein endothelial cell monolayer. *J Immunol.* 1998;161(3):1533–40. 9686621

[76] Saito H Minamiya Y Saito S: Endothelial Rho and Rho kinase regulate neutrophil migration via endothelial myosin light chain phosphorylation. *J Leukoc Biol.* 2002;72(4):829–36. 12377953

[77] Kielbassa K Schmitz C Gerke V: Disruption of endothelial microfilaments selectively reduces the transendothelial migration of monocytes. *Exp Cell Res.* 1998;243(1):129–41. 10.1006/excr.1998.4133 9716457

[78] Millán J Hewlett L Glyn M: Lymphocyte transcellular migration occurs through recruitment of endothelial ICAM-1 to caveola- and F-actin-rich domains. *Nat Cell Biol.* 2006;8(2):113–23. 10.1038/ncb1356 16429128

[79] Feng D Nagy JA Pyne K: Neutrophils emigrate from venules by a transendothelial cell pathway in response to FMLP. *J Exp Med.* 1998;187(6):903–15. 10.1084/jem.187.6.903 9500793 PMC2212194

[80] Mamdouh Z Mikhailov A Muller WA: Transcellular migration of leukocytes is mediated by the endothelial lateral border recycling compartment. *J Exp Med.* 2009;206(12):2795–808. 10.1084/jem.20082745 19887395 PMC2806621

[81] Baluk P Bolton P Hirata A: Endothelial gaps and adherent leukocytes in allergen-induced early- and late-phase plasma leakage in rat airways. *Am J Pathol.* 1998;152(6):1463–76. 9626051 PMC1858452

[82] McDonald DM Thurston G Baluk P: Endothelial gaps as sites for plasma leakage in inflammation. *Microcirculation.* 1999;6(1):7–22. 10.1111/j.1549-8719.1999.tb00084.x 10100186

[83] Rosengren S Ley K Arfors KE: Dextran sulfate prevents LTB _4_-induced permeability increase, but not neutrophil emigration, in the hamster cheek pouch. *Microvasc Res.* 1989;38(3):243–54. 10.1016/0026-2862(89)90003-4 2481803

[84] Gawlowski DM Benoit JN Granger HJ: Microvascular pressure and albumin extravasation after leukocyte activation in hamster cheek pouch. *Am J Physiol.* 1993;264(2 Pt 2):H541–6. 7680539 10.1152/ajpheart.1993.264.2.H541

[85] Kim MH Curry FR Simon SI: Dynamics of neutrophil extravasation and vascular permeability are uncoupled during aseptic cutaneous wounding. *Am J Physiol Cell Physiol.* 2009;296(4):C848–56. 10.1152/ajpcell.00520.2008 19176758 PMC2670654

[86] Valeski JE Baldwin AL: Effect of early transient adherent leukocytes on venular permeability and endothelial actin cytoskeleton. *Am J Physiol.* 1999;277(2 Pt 2):H569–75. 10444481 10.1152/ajpheart.1999.277.2.H569

[87] Heemskerk N Schimmel L Oort C: F-actin-rich contractile endothelial pores prevent vascular leakage during leukocyte diapedesis through local RhoA signalling. *Nat Commun.* 2016;7:10493. 10.1038/ncomms10493 26814335 PMC4737874

[88] Goerge T Ho-Tin-Noe B Carbo C: Inflammation induces hemorrhage in thrombocytopenia. *Blood.* 2008;111(10):4958–64. 10.1182/blood-2007-11-123620 18256319 PMC2384127

[89] Boulaftali Y Hess PR Getz TM: Platelet ITAM signaling is critical for vascular integrity in inflammation. *J Clin Invest.* 2013;123(2):908–16. 10.1172/JCI65154 23348738 PMC3561801

[90] Gros A Syvannarath V Lamrani L: Single platelets seal neutrophil-induced vascular breaches via GPVI during immune-complex-mediated inflammation in mice. *Blood.* 2015;126(8):1017–26. 10.1182/blood-2014-12-617159 26036804

[91] Nourshargh S Hordijk PL Sixt M: Breaching multiple barriers: leukocyte motility through venular walls and the interstitium. *Nat Rev Mol Cell Biol.* 2010;11(5):366–78. 10.1038/nrm2889 20414258

[92] Sorokin L: The impact of the extracellular matrix on inflammation. *Nat Rev Immunol.* 2010;10(10):712–23. 10.1038/nri2852 20865019

[93] Rowe RG Weiss SJ: Breaching the basement membrane: who, when and how? *Trends Cell Biol.* 2008;18(11):560–74. 10.1016/j.tcb.2008.08.007 18848450

[94] Wang S Voisin MB Larbi KY: Venular basement membranes contain specific matrix protein low expression regions that act as exit points for emigrating neutrophils. *J Exp Med.* 2006;203(6):1519–32. 10.1084/jem.20051210 16754715 PMC2118318

[95] Wu C Ivars F Anderson P: Endothelial basement membrane laminin alpha5 selectively inhibits T lymphocyte extravasation into the brain. *Nat Med.* 2009;15(5):519–27. 10.1038/nm.1957 19396173

[96] Proebstl D Voisin MB Woodfin A: Pericytes support neutrophil subendothelial cell crawling and breaching of venular walls *in vivo*. *J Exp Med.* 2012;209(6):1219–34. 10.1084/jem.20111622 22615129 PMC3371725

[97] Mulero V Sepulcre MP Rainger GE: Editorial: Neutrophils live on a two-way street. *J Leukoc Biol.* 2011;89(5):645–7. 10.1189/jlb.0111013 21532050

[98] Nourshargh S Renshaw SA Imhof BA: Reverse Migration of Neutrophils: Where, When, How, and Why? *Trends Immunol.* 2016;37(5):273–86. 10.1016/j.it.2016.03.006 27055913

